# Curcumin and Its Derivatives in Hepatology: Therapeutic Potential and Advances in Nanoparticle Formulations

**DOI:** 10.3390/cancers17030484

**Published:** 2025-02-01

**Authors:** Ersin Karatayli, Shifana C. Sadiq, Jörn M. Schattenberg, Stephan Grabbe, Bernhard Biersack, Leonard Kaps

**Affiliations:** 1 Department of Medicine II, Saarland University Medical Center, Saarland University, 66421 Homburg, Germany; ersin.karatayli@uks.eu (E.K.); shifanacsadiq@gmail.com (S.C.S.); joern.schattenberg@uks.eu (J.M.S.); 2 Department of Dermatology, University Medical Center of the Johannes Gutenberg-University, 55128 Mainz, Germany; stephan.grabbe@unimedizin-mainz.de; 3 Organic Chemistry Laboratory, University of Bayreuth, 95440 Bayreuth, Germany

**Keywords:** natural compounds, tumor microenvironment, *Curcuma longa*, hepatocellular carcinoma, cancer, derivatives of natural compounds, nanocarriers

## Abstract

Curcumin, a plant-derived polyphenol, shows promise in hepatology for treating both malignant and non-malignant liver diseases and a subset of extrahepatic cancers. Curcumin has hepatoprotective, anti-inflammatory, antifibrotic, and antiproliferative properties, as is evident in preclinical and clinical studies. This highlights its potential as an adjunct to established cancer therapies, especially in the context of hepatocellular carcinoma and secondary liver malignancies. Curcumin also demonstrates potential in metabolic dysfunction-associated steatotic liver disease (MASLD), owing to its antifibrotic and lipid-lowering effects. However, its clinical use is limited, relating to its poor bioavailability and rapid metabolism. Nanotechnology, including liposomal and polymeric carriers, alongside synthetic curcumin derivatives, offers strategies to enhance the bioavailability and pharmacokinetic properties. We propose to revisit the use of curcumin in nanoparticle preparations in chronic liver disease and summarize current evidence in this review article.

## Simple Summary

Liver diseases are a significant contributor to global mortality, representing an escalating public health concern worldwide. Natural compounds, particularly herbal derivatives, remain a primary source for therapeutic agents in liver disease management. Among these, curcumin, the principal active compound in turmeric, has demonstrated substantial therapeutic potential. However, its clinical application is limited by poor pharmacokinetics, including low bioavailability and rapid metabolic degradation. This review provides a comprehensive analysis of nanoparticle-based formulations designed to enhance the pharmacokinetics of curcumin for the treatment of non-malignant liver diseases. By examining advancements in nanomedicine, such as liposomal and polymeric delivery systems for curcumin derivatives, this study highlights innovative strategies to address the critical challenges associated with curcumin-based therapies. The insights presented here consolidate the current knowledge on curcumin’s therapeutic potential in liver diseases, with a focus on its incorporation into nanocarriers, providing a robust foundation for future research into this promising herbal compound.

## 1. Introduction

The liver, the largest solid organ in the human body, plays a vital role in the metabolism of amino acids, carbohydrates, lipids, vitamins, and the synthesis of proteins. Apart from its metabolic functions, it also acts as a primary filter, removing pathogens and exogenous antigens from the circulation. All these metabolic and immunological activities of the liver increase exposure to neo-antigens, necessitating unique immune mechanisms to balance tolerance and responsiveness [1].

Chronic liver disease (CLD) is characterized by inflammation-driven fibrogenesis, which can lead to a progressive decline in liver function. Hepatic inflammation, parenchymal destruction, and regeneration eventually lead to end-stage liver disease (ESD) and cirrhosis [2]. A lethal complication of cirrhosis is hepatocellular carcinoma (HCC), the most prevalent type of primary liver cancer [3]. Despite recent progress with immunotherapy, the prognosis of patients with HCC is still dismal due to the tumor’s inherited resistance to chemotherapy [4].

Plants are a rich source for modern drug development [5]. It is estimated that approximately 11% of the 252 essential drugs recognized by the World Health Organization (WHO) are exclusively plant-derived. One such example is curcumin, the principal curcuminoid, chemically known as the polyphenol diferuloylmethane, found in turmeric (Curcuma longa). In plants, curcumin plays a protective role, shielding them from oxidative stress and microbial threats [6].

**Figure 1:**
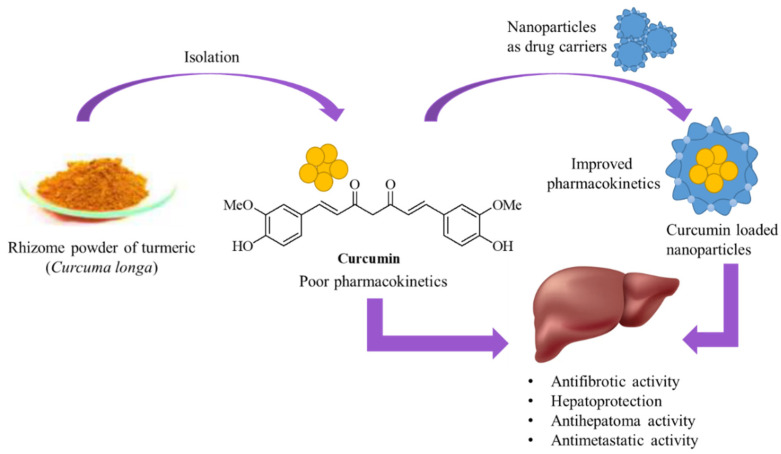
Main functions of curcumin and curcumin nanoparticle.

Curcumin has been well-recognized for centuries for its therapeutic value, especially in traditional Chinese medicine and Ayurveda, as an herbal remedy. Beyond its medicinal applications, curcumin is commonly used as a spice and in skincare formulations in the Indo-Asia region. Beyond its traditional uses, curcumin has gained increasing attention in modern Western medicine. Recent advancements in nanotechnology have facilitated the encapsulation of curcumin in nanoparticles, which could enhance the bioavailability of curcumin and, thereby, improve its therapeutic efficacy for treating various diseases (Figure 1).

Growing evidence has proven curcumin’s broad spectrum of pharmacological benefits, such as anti-inflammatory [7], antioxidant [8,9], antisteatotic [10,11] antifibrotic, and antitumor activity, rendering curcumin a valuable compound to treat malignant and non-malignant liver disease [11,12,13].

Despite all the positive effects, there are also safety concerns regarding the consumption of curcumin, as excessive doses may cause drug-induced liver injury (DILI) [14].

In this review, we provide a comprehensive overview of curcumin and its pharmacological value in hepatology.

## 2. Antitumor Properties of Curcumin in Hepatocellular Carcinoma

**Figure 2:**
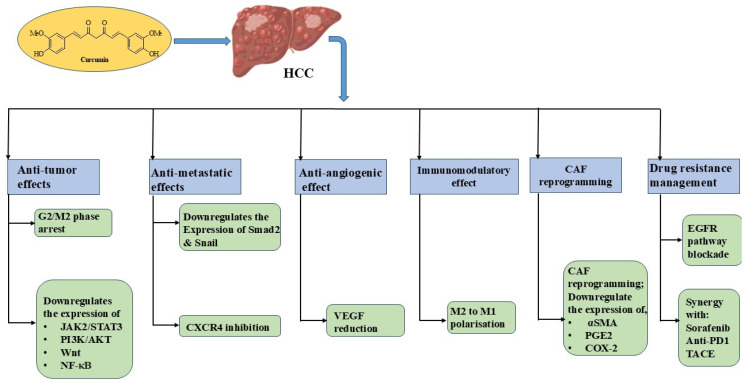
Schematic representation of tumor suppressive mechanism induced by curcumin exhibits multiple therapeutic actions, including antitumor effects through G2/M2 phase arrest and the downregulation of JAK2/STAT3 (Janus kinase 2/signal transducer and activator of transcription 3), PI3K-AKT (phosphatidylinositol 3-kinase-protein kinase B), Wnt, and NF-κB (nuclear factor kappa-light-chain-enhancer of activated B cells) pathways. Antimetastatic effects are achieved via downregulation of Smad2 and Snail, along with CXCR4 (C-X-C motif chemokine receptor 4) inhibition. Antiangiogenic effects occur through VEGF (vascular endothelial growth factor) reduction. Immunomodulatory effects involve M2 to M1 macrophage polarization. Cancer-associated fibroblast (CAF) reprogramming occurs through downregulation of α-SMA (alpha-smooth muscle actin), PGE2 (prostaglandin E2), and COX-2 (cyclooxygenase-2). Drug resistance management is facilitated through EGFR (epidermal growth factor receptor) pathway blockade and synergy with sorafenib, anti-PD1 (programmed cell death protein 1), and TACE (transarterial chemoembolization).

Hepatocellular carcinoma (HCC) is the most prevalent form of primary liver cancer. Despite recent advancements in therapeutic strategies, including immunotherapy, the median overall survival rate of patients with advanced stage of HCC is limited to 2 years. This poor prognosis in the advanced stage suggests the urgent need of pharmacological agents, using different mechanisms, in addition to established regimes [15,16]. Curcumin might offer an additional therapeutic benefit to established regimens, as it inhibits HCC through multiple mechanisms (Figure 2). First, it suppresses cell proliferation by G2/M phase cell cycle arrest in HCC [17]. Second, growth inhibition and apoptosis induction by curcumin occurs by the downregulation of JAK2/STAT3 and PI3K/AKT signaling [9,18,19,20]. Third, curcumin-mediated HCC cell death involves other forms of programmed cell death, such as pyroptosis, ferroptosis, cuproptosis, necroptosis, and autophagy [11,13,16,21]. Fourth, curcumin regulates the expression of various miRNAs in HCC, leading to cell death [22,23,24]. Fifth, tumor-related cell proliferation and inflammation are inhibited by interfering with the Wnt signaling and NF-κB pathways [25,26,27]. In addition to its effect on established tumors, curcumin inhibits metastasis formation and epithelial–mesenchymal transition (EMT) by the suppression of Smad2 and Snail [28]. Beyond this, curcumin exerts a distinct effect on the surrounding tumor tissue, the tumor microenvironment (TME). Curcumin has an antiangiogenic effect, hampering the supply of oxygen and nutrients to the tumor [29,30]. In this regard, VEGF is one of the most proangiogenic factors, which is mainly responsible for neovascularization in tumors [31]. Curcumin decreases VEGF serum levels in different murine models of HCC, thereby inhibiting neovascularization and restraining tumor growth and HCC development [32]. Curcumin also possesses an immunomodulatory effect in the TME. It polarizes M2 tumor-associated macrophages towards the M1 antitumor phenotype and impedes the recruitment and aggregation of myeloid-derived suppressor cells [33]. In addition to its effect on immune cells, curcumin also affects the stromal compartment in the TME, which represents the largest portion of the TME [34]. Similar to macrophages, cancer-associated fibroblasts (CAFs) are reprogrammed by curcumin towards an immunocompetent phenotype [35]. In the study, CAFs were extracted from breast cancer tissue treated with curcumin ex vivo and, subsequently, cocultured with corresponding peripheral blood mononuclear cells (PBMCs). The curcumin treatment reduced CAF-related markers, such as α-SMA, COX-2, and the production of PGE2 in CAFs, as well as markers for immunosuppression, e.g., FoxP3, TGF-β, IL-10, and IL-4, while IFN-γ production was upregulated in PBMCs.

Curcumin has exhibited the potential to overcome drug resistance in HCC, which is the most significant challenge in HCC therapy [36]. For instance, resistance to the second-line drug Lenvatinib has been overcome by curcumin via blocking the EGFR pathways, a common resistance mechanism in HCC [37].

In combination with checkpoint inhibitors, curcumin revealed synergistic effects with the anti-PD-1 antibody and sorafenib, which are used for first- and second-line treatment in HCC [38,39].

In addition to systemic therapy, curcumin combined with piperine and taurine augmented the anticancer activity of transarterial chemoembolization therapy (TACE) in HCC patients, which is an ablation procedure and the current standard therapy for patients with intermediate-stage HCC [40].

Curcumin also protects against secondary liver cancer, where metastases originate from colon or gastric cancer [41]. Although the exact molecular mechanism was not revealed, the study by Herrero de la Parte et al. suggests that curcumin treatment in CC531 cells inhibited cell migration and invasiveness in wound healing assays [42]. While Gu et al. proposed a mechanism for the metastasis restriction of primary gastric cancer cells through a reduced number of circulating tumor cells due to curcumin-mediated inhibition of CXCR4 expression, a receptor necessary for tumor cells to home secondary organs [41]. However, its therapeutic value as an antimetastatic agent may extend beyond gastrointestinal cancers, as a synthetic curcuminoid suppressed lung and liver metastases in a murine B16F10 melanoma model [43].

## 3. Derivatives of Curcumin with Improved Antitumor Properties

**Figure 3:**
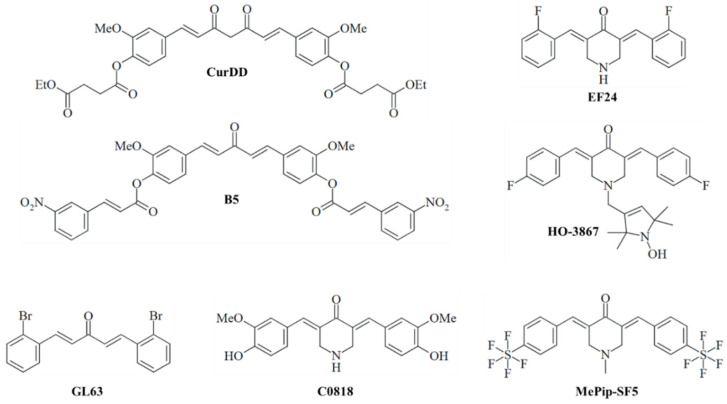
Structures of (semi-)synthetic curcumin derivatives with improved activities against liver cancers and metastases.

Numerous synthetic and semisynthetic derivatives of curcumin have been developed to improve its antitumor properties. These derivatives have demonstrated improved pharmacological activities, including increased bioavailability and potency. Several derivatives have exhibited promising activities against liver cancers and hepatic metastases, which are presented in the following (Figure 3).

### 3.1. Curcumin Diethyl Disuccinate (CurDD)

Curcumin diethyl disuccinate (CurDD) is an ester prodrug of curcumin with improved chemical stability at pH 7.4 and enhanced transmembrane transport across Caco-2 (human colon cancer cells) monolayers, leading to a higher amount of the bioavailable drug fraction. CurDD increased apoptosis induction in HepG2 hepatoma cells by the suppression of Bcl-2 and the upregulation of Bax, followed by the activation of caspases 3 and 9 [44].

### 3.2. Monocarbonyl Analogs of Curcumin (MACs)

Monocarbonyl analogs of curcumin (MACs) are an especially prolific class of anticancer active curcuminoids [45]. These include B5, EF24, HO-3867, GL63, C0818, and MePip-SF5.

#### 3.2.1. B5

The 3-nitrocinnamate-modified monocarbonyl derivative B5 was more active than curcumin against HepG2 cells and induced caspase 3-dependent apoptosis by downregulating Bcl-2 and AKT. In addition, B5 inhibited the migration of HepG2 cells [46].

#### 3.2.2. GL63

The brominated MAC GL63 inhibited HCC growth in vitro and in vivo by the suppression of the circRNA zinc finger protein 83 (circZNF83) upon the GL63-induced activation of miR-324-p followed by the inhibition of CDK16 expression and downregulation of the JAK2/STAT3 signaling pathway [47].

#### 3.2.3. EF24

MACs derived from piperidin-4-ones have turned out to be particularly promising based on their improved anticancer activities and bioavailabilities. EF24 [3,5-bis-(2-fluorobenzylidene)-4-piperidone] is the most prominent piperidin-4-one-based example with pronounced antitumor properties [48]. Of note, EF24 was successfully incorporated into drug delivery systems, which include pegylated liposomes as hydroxypropyl-β-cyclodextrin-encapsulated compounds in liposomes and as a covalent conjugate with the coagulation factor VIIA [49,50,51]. In terms of antihepatoma activity, EF24 exhibited anti-invasive and antimigratory activities against HCCLM-3 and HepG2 cells (HCC cells), in accordance with suppressed filopodia formation on the surface of treated cells. The inhibition of Src phosphorylation by EF24 was identified as the underlying mechanism of action [52]. Moreover, EF24 inhibited HCC growth in vitro and in vivo associated with strong apoptosis induction (Bax and caspase 3 activation, Bcl-2 suppression) and cell cycle arrest in the G2/M phase (cyclin B1 and Cdc downregulation, p53 and p21 upregulation) [53]. EF24 also overcame sorafenib resistance in hypoxic HCC by the promotion of the VHL-mediated degradation of HIF-1α [54].

#### 3.2.4. HO-3867

HO-3867 is a modified EF24 derivative (with a different position of the fluoro substituent and a piperidin-4-one N-modified with a 1-hydroxy-2,2,5,5-tetramethyl-2,5-dihydro-1H-pyrrole appendage) that showed considerable p38-dependent apoptosis induction, accompanied by HO-1 and caspase activation [55].

#### 3.2.5. C0818

C0818, harboring the 3-hydroxy-4-methoxyphenyl moieties of curcumin, was more antiproliferative than curcumin in HepG2 and Sk-Hep-1 HCC cells and led to distinct apoptosis induction via the mitochondrial pathway. In addition, C0818 inhibited Hsp90 in HCC cells, leading to the proteasomal degradation of proteins in the Ras-MAPK and PI3K-AKT signaling pathways, which, as a consequence, were downregulated in C0818-treated HCC cells [56].

#### 3.2.6. MePip-Sf5

Our group has recently studied the promising anticancer effects of the pentafluorothio-substituted analog MePip-SF5 [57,58]. This compound was well-tolerated by mice bearing B16F10 murine melanoma and suppressed metastasis formation in vital organs, such as the liver and lungs [57].

## 4. Curcumin in Steatotic Liver Disease (SLD) and Lipid Metabolism

**Figure 4:**
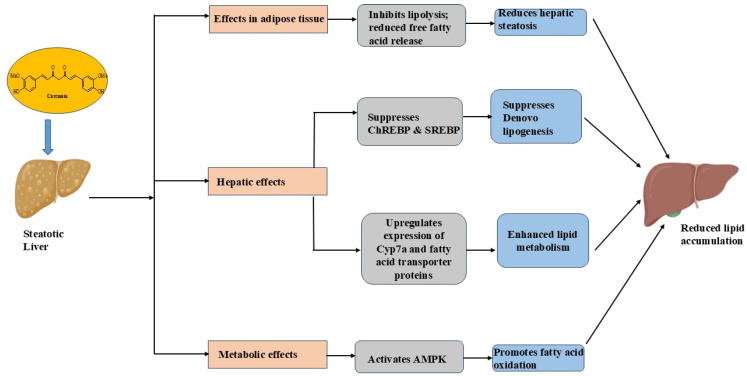
Schematic representation of the lipid-lowering activity exhibited by curcumin. The diagram illustrates how curcumin acts on the steatotic liver through multiple pathways. In adipose tissue, curcumin leads to inhibited lipolysis and reduced free fatty acid release, ultimately reducing hepatic steatosis; the downregulation of CHREBP (carbohydrate-responsive element-binding protein) and SREBP (sterol regulatory element-binding protein) suppresses de novo lipogenesis; the upregulation of CYP7a (cytochrome P450 7A1) and fatty acid transporter proteins enhances lipid metabolism; the activation of AMPK (5′ adenosine monophosphate-activated protein kinase) promotes fatty acid oxidation. These combined mechanisms result in reduced lipid accumulation in the liver.

Steatotic liver disease (SLD) is an umbrella term that encompasses a range of liver diseases characterized by the accumulation of fat in hepatocytes. SLD is divided into three main categories: metabolic dysfunction-associated steatotic liver disease (MASLD), alcohol-related liver disease (ALD), and the mixed entity metALD [58]. MASLD refers to fat accumulation in the liver not caused by alcohol consumption and can be either noninflammatory or inflammatory. It affects approximately 30% of the global population, with incidence rates rising, particularly in Western countries, due to factors such as a high-caloric diet, sedentary lifestyles, and increasing rates of obesity [59]. The inflammatory subtype, metabolic dysfunction-associated steatohepatitis (MASH), involves liver inflammation and cell damage. Over time, inflammation leads to progressive fibrosis (scarring) that can progress to cirrhosis, the end-stage of all chronic liver diseases associated with organ failure and other life-threatening complications. Curcumin demonstrates promising therapeutic efficacy against MASH and holds promise as an adjuvant therapy (Figure 4) [34]. Curcumin has also been studied in patients with SLD progression and demonstrated a reduction in glycerides and waist circumferences [60].

The lipid-lowering effect of curcumin has gained attention for its potential in managing conditions related to dyslipidemia [61]. By reducing elevated levels of cholesterol and triglycerides, curcumin acts as an effective antilipidemic agent, optimizing lipid homeostasis [62]. This lipid-lowering effect of curcumin contributes to the prevention and treatment of fatty liver diseases [63]. By inhibiting lipolysis, curcumin prevents free fatty acid (FFA) release from adipose tissue, reducing hepatic steatosis. It also suppresses factors like carbohydrate response element binding protein (ChREBP) and sterol regulatory element binding protein (SREBP1c), which decrease de novo lipogenesis (DNL). This makes curcumin a promising therapeutic option in addressing metabolic disorders, such as MASLD or MASH [64].

Curcumin inhibits the activity of Acyl-CoA: cholesterol acyltransferase (ACAT) and activates hormone-sensitive lipase aiding lipid mobilization. Additionally, curcumin increases the secretion of bile acids and upregulates the expression of hepatic cholesterol 7a-hydroxylase (Cyp7a) and fatty acid transporter proteins, enhancing bile production and lipid metabolism [65].

Curcumin also impacts lipid homeostasis by reducing the expression of LDL receptors through the activation of peroxisome proliferator-activated receptor γ (PPAR γ) in hepatic stellate cells [66]. At the same time, it upregulates LDL receptors and increases LDL uptake in macrophages [67]. Macrophages can intake oxidized lipoproteins LDL and VLDL through diverse mechanisms, such as micropinocytosis and phagocytosis, despite the scavenger receptor-mediated pathways, such as LOX-1, SR-A1, CD36, and SR-B1 [68]. Furthermore, curcumin boosts AMP-activated protein kinase (AMPK) activity, promoting fatty acid oxidation and reducing malonyl-CoA expression [67]. Musso et al. proved the therapeutic effect of phytosomal curcumin (Meriva^®^, a curcumin–phosphatidylcholine complex, containing 20% curcuminoids) for MASH in a double-blind, placebo-controlled, randomized clinical trial [69]. The authors state that MASH resolution occurred in 62% of patients treated with curcumin versus 12% of patients in the placebo arm. Further, histological fibrosis regression (at least 1 histological stage) was achieved in 50% in the curcumin versus 8% in the placebo arm. The histological improvement was independent of weight loss, and no differences in diet between the groups were observed. Meriva^®^ also revealed chemopreventive effects on hepatitis B virus-related HCC in transgenic mice [30]. In another interesting double-blind, randomized trial conducted by Y He et al., patients with fatty liver disease received 500 mg per day curcumin or placebo over 24 weeks [70]. Hepatic fat content was assessed by FibroTouch-based controlled attenuation parameters (CAPs), a non-invasive ultrasound-based method. Further, microbial composition and bile acid metabolites were analyzed using 16S rRNA sequencing and metabolomics. Curcumin consumption significantly reduced CAP value compared to placebo (−17.5 dB/m; 95% confidence interval [CI]: −27.1, −7.8 dB/m; *p* < 0.001) and also induced a reduction in weight (−2.6 kg; 95% CI: −4.4, −0.8 kg; *p* < 0.001) compared to the placebo group, which is in contrast to the results presented by G Musso et al. [69], where patients showed histological improvement without weight loss. In addition, blood parameters for free fatty acid (*p* = 0.004), triglycerides (*p* < 0.001), fasting blood glucose (*p* = 0.038), and hemoglobin A1c (*p* = 0.019) significantly improved. The treatment also had an effect on the gut microbiota, decreasing the firmicutes-to-bacteroidetes ratio. Conclusively, curcumin is an interesting phytopharmaceutical drug for MASH, the therapeutic benefits of which could be enhanced when packaged in nanoparticles.

## 5. Pharmacokinetics of Curcumin

Curcumin has gained extensive attention for its remarkable therapeutic properties, including anti-inflammatory, antioxidant, anticancer, and hepatoprotective effects. However, despite its immense potential, curcumin faces significant pharmacokinetic challenges that hinder its clinical application.

Following oral administration, curcumin demonstrates minimal absorption in the gastrointestinal tract. Studies have reported that up to 90% of the ingested compound is eliminated through fecal excretion, leaving only a small fraction available for systemic circulation [71]. This issue is further compounded by its extensive first-pass metabolism in the liver and plasma, where it undergoes glucuronidation and reduction, drastically reducing its active concentrations in the bloodstream [72].

Additionally, curcumin is chemically unstable under alkaline conditions, degrading rapidly into metabolites, such as ferulic acid, feruloyl methane, and vanillin, within approximately 30 min [73]. While it exhibits greater stability in acidic environments, its solubility remains limited across neutral and acidic pH levels, adding to the complexity of its bioavailability challenges [74]. Despite these limitations, curcumin is recognized as Generally Recognized as Safe (GRAS) by the FDA, and clinical studies have shown that, even at high doses of up to 12 g/day, it is well tolerated without causing significant adverse effects [75].

To overcome these pharmacokinetic obstacles, researchers have pursued various strategies aimed at enhancing curcumin’s bioavailability and therapeutic efficacy. One of the most widely studied approaches is coadministration with piperine, a natural compound found in black pepper, which inhibits glucuronidation processes and significantly increases curcumin’s plasma concentrations [72]. In addition, advanced drug delivery systems, such as liposomal formulations, curcumin nanoparticles, and phospholipid complexes, have been developed to protect curcumin from rapid degradation. All this improves its absorption and stability and facilitates its transport across biological membranes, thereby enhancing its systemic availability [73,76].

Furthermore, the synthesis of structural analogs, such as EF-24, has demonstrated promising results in improving the half-life and bioavailability of curcumin, thus offering a potential solution to its pharmacokinetic challenges [74]. Despite these advancements, the clinical efficacy of these enhanced formulations compared to standard curcumin remains an area requiring further investigation. Future research is essential to optimize these delivery systems and analogs, thereby establishing their long-term safety and efficacy and determining the most effective dosing strategies to achieve therapeutic concentrations [71,77]. While significant progress has been made, realizing the full clinical potential of curcumin continues to be a multidisciplinary challenge that combines pharmaceutical innovation with rigorous clinical evaluation.

Nanocapsulation with carriers like liposomes or polymer-based nanoparticles can improve the poor bioavailability and solubility of curcumin to certain extent. These nanoformulations protect curcumin from rapid degradation in the gastrointestinal tract and enzymatic metabolism, while enabling controlled release and targeted delivery to specific tissues. Additionally, modern nanomanufacturing techniques can address production challenges by improving batch consistency and stability. By following the DELIVER framework’s principles for nanomedicine development, researchers can systematically address regulatory requirements and enhance the likelihood of successful clinical translation of curcumin-based therapeutics [78]. Thus, nanocarriers can significantly enhance the pharmacokinetics of curcumin by improving its stability and bioavailability to a great extent, thereby optimizing its therapeutic application.

## 6. Nanocarriers Improve Pharmacokinetics of Curcumin

The therapeutic application of curcumin is critically limited because of its hydrophobic nature, unstable chemical structure, rapid hydrolysis, and poor absorption. Chemical modification can address some of these limitations, but guided organotropism remains challenging. There have been efforts to overcome these challenges by the design and synthesis of nanocarrier liposomes to establish better permeability, higher absorption, greater stability for longer circulation, and improved resistance to the rapid metabolic hydrolysis of curcumin in the body, improving hepatic delivery for the therapy of (non-)malignant liver disease (Table 1).

In a set of double-blind, randomized, placebo-controlled clinical trials, Jazayeri-Tehrani et al. demonstrated that curcumin, delivered via poly(lactic-co-glycolic acid) (PLGA) nanoparticles, effectively improves inflammatory markers and nesfatin (anorexigenic neuropeptide) levels and reduces appetite in obese patients with nonalcoholic fatty liver (previous nomenclature of fatty liver disease) [79,80,93]. Then, compared to the placebo, significant increases in high-density lipoprotein (HDL) and quantitative insulin sensitivity check index (QUICKI) and significant decreases in fatty liver degree, liver transaminases, waist circumference, fasting blood sugar and insulin (FBS and FBI), triglycerides (TG), total cholesterol (TC), low-density lipoprotein (LDL), homeostasis model assessment insulin resistance (HOMA-IR), tumor necrosis factor-α (TNF-α), high sensitive c-reactive protein (hs-CRF), and interleukin-6 (IL-6) were reported in these clinical trials.

A study by Singh et al. compared the efficacy of the well-established hepatoprotectant, silymarin, in combination with free curcumin versus curcumin packaged into solid lipid nanoparticles (C-SLNs) in a rat model of CCl_4_-induced hepatic injury [81]. The histopathological examination of liver tissues in mice treated with vehicle control (VC; no treatment), free curcumin (FC), SILY (sylmarin), and C-SLNs showed that C-SLNs were superior to improve steatosis and to ameliorate inflammation. Highly elevated ALT levels due to CCl_4_ administration decreased significantly (*p* < 0.001) compared to VC-, FC-, and SILY- treated groups (2.63-, 1.87-, and 1.97-fold reductions in C-SLNs, FC, and SILY groups, respectively). A similar effect was also observed for AST levels. Moreover, a significant attenuation in oxidative stress was achieved by C-SLN treatment compared to FC and SILY treatment. The malondialdehyde (MDA) content, which is a measure of lipid peroxidation, increased by 482.88 ± 58.83% after the CCl_4_ challenge, as compared to the VC group. Treatment with C-SLNs resulted in an inhibition of 72.05 ± 1.14%, while inhibition levels were 58.15 ± 2.03 for FC and 51.46 ± 1.11% for SILY. Similar effects were also observed in superoxide dismutase (SOD) and glutathione (GSH) levels, suggesting the improved attenuation of oxidative stress in C-SLN group. Moreover, the inflammatory response was assessed by TNFα levels, which was significantly elevated after the CCl_4_ challenge. The decrease in TNFa levels in the C-SLN group (6.28-fold) was also significantly greater (*p* < 0.05) than those observed in the SILY group (5.02-fold) and FC group (4.68-fold), suggesting a better resolution of inflammation for curcumin loaded in nanoparticles.

A recent experiment by Wu et al. compared the efficacy of free curcumin, curcumin-loaded poly(lactic-co-glycolic acid) (PLGA) nanoparticles (CPNPs), and platelet membrane-coated, curcumin-loaded CPNPs (PCPNPs) in HepG2 tumor bearing BALB/c null mice. Mice were treated by intravenous administration every 2 days for a total of seven times. The higher resistance of curcumin to hydrolysis was evident for PCPNPs with a prolonged circulation time, which was shown by significantly longer mean residence time (MRT) and lower clearance (Cl). Area under drug concentration–time curve was found to be 3.44-fold and 1.29-fold higher than that of free curcumin and CPNPs, respectively. Moreover, tumor-targeting ability was higher for the nanoparticle formulation, as shown by significantly increased accumulation in tumors. PCPNPs also had the highest potential of antitumor activity with a tumor growth inhibition of 83.4% compared to that of the CPNPs (57%) and free curcumin (28.9%) [83]. In another study, activated hepatic stellate cells (aHSC) and hepatoma cells were targeted using modified mixed nanoliposomes co-encapsulating berberin and curcumin. Hyaluronic acid (HA)-modified nanoparticles targeted CD44, which is overexpressed by aHSC. Glycyrrhetinic acid (GA)-modified carriers, on the other hand, can accumulate in cancer cells by GA receptor-mediated internalization. H22 cell-bearing HCC mice treated with the modified nanoliposomes carrying curcumin and berberine (CUR-GL/BBR-HL) showed lower tumor volume than the free drug and non-modified liposomal formulations with a tumor growth inhibition rate of 68.56%, which is 1.34- and 1.85-fold of non-modified liposomal carriers (CUR-L/BBR-L) (51.15%) and free drugs (CUR + BBR) (36.93%), respectively. To further detect the antihepatoma efficacy, primary liver cancer was induced using orthotopic-transplanted mice. The combined therapy significantly decreased the size and number of tumors, together with lower levels of extracellular matrix deposition (ECM). Moreover, CD31 immunohistochemistry (IHC) has shown that CUR-GL/BBR-HL effectively inhibits neovascularization in the tumor microenvironment [86].

The liposomal formulations of curcumin were frequently studied in liver cancer models, and promising results were observed for curcumin formulations with modified liposomes, such as galactose–morpholine- and hyaluronic acid–glycyrrhetinic acid-modified liposomes [86,87,94,95]. In one such study, Yang et al. successfully integrated galactose group that recognizes asialoglycoprotein receptor (ASGPR) and morpholine group-targeting lysosomes into nanoliposomes, which were used for the hepatocyte-targeted delivery of curcumin [87]. In this context, ASGPR, which is primarily expressed on hepatocytes and hepatoma cells, has long been studied as a promising candidate for receptor-mediated hepatocyte targeting in this regard [96]. The tumor inhibition of dual-targeted nanoliposomal formulation (Gal-Mor-LPs) was shown to be improved compared to free curcumin, curcumin loaded in unmodified liposomes (LPs), and galactose-modified liposomes (Gal-LPs). The cellular uptake of Gal-Mor-LPs was approximately 1.5 times higher than that of Gal-LPS both in vitro and in vivo. Gal-Mor-LPs treatment resulted in a superior antitumor effect, as revealed by a smaller tumor volume and weight compared to controls. Moreover, the Gal-Mor-LPS group showed more necrosis in the histopathological analysis [87].

The polymeric formulations of curcumin with PLGA and polyvinylpyrrolidone were also described for liver cancer treatment [83,97,98,99]. Notably, a polymeric coformulation of curcumin and sorafenib efficiently suppressed both growth and metastasis formation in a murine HCC model (established by the subcoutenaous injection of human HCC cell lines with high metastatic potential, namely MHCCLM3-RFP, in athymic BALB/c nu/nu mice) in vivo [100]. Antihepatoma curcumin formulations using polysaccharides, such as chitosan and angelica polysaccharide, were also reported [38,101]. Recently, a sophisticated formulation of a europium metal organic framework coated with a lactoferrin-modified, dextran-based chitosan enabled the efficient HCC-targeting of curcumin [102]. Thus, the pharmacokinetics and therapeutic efficacy of curcumin could be improved in (non-)malignant liver disease when loaded with nanoparticles and liposomal formulations in vivo.

## 7. Limitations of Curcumin Nanoparticles

Even though curcumin nanoformulations hold promise in addressing the intrinsic limitations of curcumin, they face several challenges that hinder their broad clinical application. A major limitation is the lack of clinical data, as most evidence supporting their efficacy is derived from preclinical studies, highlighting a significant gap in translational research [103].

Additionally, the poor bioavailability of curcumin remains a persistent issue, attributed to its hydrophobic nature, rapid metabolism, and short systemic half-life, which collectively restrict its therapeutic efficacy. Notably, even in nanoparticle formulations, curcumin exhibits reduced bioavailability, posing a critical barrier to better therapeutic efficacy [104].

Furthermore, curcumin’s high susceptibility to photodegradation and its instability in alkaline environments complicate its formulation, storage, and long-term application [105]. In oncology, the restricted loading capacity of nanoparticles of curcumin makes curcumin low potent compared to conventional chemotherapeutic agents [106]. The curcumin formulations could loss functionality over time which marks a significant concern [107]. Additionally, the long-term safety profiles of curcumin nanoparticles remain inadequately understood. Potential risks, such as organ accumulation or the cytotoxic effects associated with the nanocarriers, necessitate rigorous investigation to ensure the safety of curcumin-based therapies [108]. In this context, polyethylene glycol (PEG) chains, widely employed in nanoparticles for their protein-repellent and immune system “stealth” properties, carry the potential risk of forming PEG vacuoles in the kidneys, with uncertain long-term health consequences. This concern underscores the advantage of using nanoparticles coated with biocompatible alternatives, such as sarcosine, a naturally occurring amino acid, as a probably safer option [109,110].

These multifaceted challenges emphasize the need for innovative and comprehensive approaches to enhance the pharmacokinetics, stability, and clinical reliability of curcumin-based therapies and curcumin nanoformulations, paving the way for their successful translation into clinical practice.

## 8. Conclusions

In conclusion, curcumin is a multifaceted plant-derived drug with significant potential in hepatology, particularly in the treatment of both malignant and non-malignant liver diseases but also other malignant diseases outside the liver. Its hepatoprotective, anti-inflammatory, antifibrotic, and anticancer properties, demonstrated in preclinical and clinical studies, show its versatility as a therapeutic option. Curcumin’s ability to modulate crucial signaling pathways, enhance immune responses, and impede angiogenesis and metastasis formation renders it a promising adjunct to conventional cancer therapies, particularly in hepatocellular carcinoma (HCC) and secondary liver malignancies. Furthermore, curcumin’s impact on metabolic dysfunction-associated steatotic liver disease (MASLD), notably its antifibrotic and lipid-reducing effects, suggests its potential as a treatment strategy for metabolism-associated liver conditions, like metabolic dysfunction-associated steatohepatitis (MASH). However, despite these promising properties, the clinical application of curcumin is hampered by its poor bioavailability and rapid metabolism. Advances in nanotechnology, including liposomal and polymeric formulations, offer promising avenues to overcome these limitations and enhance curcumin’s therapeutic efficacy, especially in liver disease, where most of the nanoparticles accumulate in high doses after intravenous injection. Besides nanocarriers, synthetic curcumin derivatives have shown enhanced stability, bioavailability, and antitumor activity, presenting further opportunities for improving liver cancer treatment outcomes. Like any effective drug, possible side effects need to be taken into account. Based on the National Health and Nutrition Examination Survey (NHANES), six hepatotoxic botanical products were identified. It was found that turmeric is potent enough to cause severe and even fatal liver damage when consumed in excessive doses. Hence, there is a critical need for finding the right dose to reduce hepatotoxic risks [14]. Future clinical trials are warranted to validate the therapeutic efficacy of curcumin encapsulated in nanocarrier and curcumin derivatives, particularly in combination with existing liver disease treatments. This could pave the way for integrative treatment approaches in liver diseases, offering solutions to persistent therapeutic challenges, while addressing critical unmet clinical needs.

## Figures and Tables

**Table 1:** Selected preclinical studies of curcumin-loaded nanoparticles in liver disease.

Study	Curcumin Derivative	Liver Disease	Preclinical Models/ Clinical Models	Nanotechnology	Result	Advantages	References
Clinical	CUR	NAFLD	- NAFLD in overweight/obese patients	Nanocurcumin capsules as polylactic-co-glycolic acid (PLGA) nanoparticles provided by a company	- Improved glucose indices, lipids, inflammation, waist circumference, nesfatin, liver transaminases, and fatty liver degree. Reduced appetite	- Elevated serum nesfatin levels	[28,79,80]
Preclinical	CUR	Liver fibrosis	- CCl_4_ induced liver injury in Wistar rats	Curcumin-loaded solid lipid nanoparticles (C-SLNs)	- Reduced liver damage - Ameliorated inflammation - Reduced steatosis state - Reduced oxidative stress - Suppressed proinflammatory cytokine TNF-α	- Improved bioavailability - Enhanced therapeutic efficacy	[81]
Preclinical	CUR	Liver fibrosis	- CCl_4_ induced liver injury in C57BL/6 mice	Curcumin containing silver nanoparticles (AgNPs-curcumin)	- Prevented oxidative imbalance - Prevented hepatic dysfunction - Prevented tissue destruction - Increased antioxidant activity	- Improved dispersion - Improved bioavailability	[82]
Preclinical	CUR	HCC	- HepG2 cell line - HepG2 tumor-bearing BALB/c nude mice	Platelet membrane-coated CUR-loaded polylactic-co-glycolic acid (PLGA) nanoparticles	High anticancer efficacy without obvious toxicity	- Immune evasion - Improved accumulation at tumor sites - Prolonged circulation - Increased cellular uptake	[83]
Preclinical	C210	HCC	- Mouse hepatoma H22 cells - H22 tumor-bearing ICR mice	Redox-responsive lipidic prodrugnanodelivery system of C210 (nanoparticles with single sulfide bond, i.e., C210-S-OA nanoparticles)	Improved antitumor activity and bioavailability	- Improved accumulation at tumor sites - Prolonged circulation - Increased cellular uptake	[84]
Preclinical	CUR	HCC	- HepG2 cell line - HepG2 tumor-bearing BALB/c nude mice	Curcumin–paclitaxel lipid nanoplatform (CU-PTX-LNP platform)	- Improved antitumor activity and bioavailability	- Sustained release - Long-lasting stability - Enhanced curcumin absorption - Excellent biosafety	[85]
Preclinical	CUR (in combination with berberine)	HCC	- Dual-cell research model (SMMC7721 + LX-2) - Orthotopic tumor-bearing mice	Mixed liposomes of curcumin loaded on glycyrrhetinic acid-modified nanocarriers and berberine loaded on hyaluronic acid-modified nanocarriers (CUR-GL/BBR-HL)	- Enhanced anticancer effect	- Inhibition of hepatic stellate cell activation and ECM deposition - Inhibition of proliferation and metastasis of HCC	[86]
Preclinical	CUR	HCC	- HepG2, Huh7 cell lines - Tumor-bearing Kunming mice injected with H22 cells (mouse hepatoma cell line)	Galactose–morpholine modified liposomes loaded with curcumin (Gal-Mor-LPs)	- Enhanced tumor inhibition efficacy	- Sustained drug release - Dual hepatic and lysosomal targeting - Enhanced cellular uptake	[87]
Preclinical	Curcumin derivative (CDF)	HCC	- HepG2 cell line - HepG2 tumor-bearing mice	Curcumin loaded on nanoscale G4 polyamidoamine (PAMAM) dendrimers anchored to galactosamine(PAMAM-Gal conjugate)	- Reduced cytotoxicity - Antitumor response	- Enhanced aqueous solubility - Better accumulation at tumor site - Selective targeting - Increased drug loading	[88]
Preclinical	CUR	HCC	- DEN-induced hepatocellular model in Wistar albino rats	- Biodegradable liver-specific pullulan acetate nanoparticles (PAC)	- Reduced liver enzyme levels - Increased levels of non-enzymatic antioxidants	- Improved encapsulation efficiency - Enhanced hepatoprotective agent - Enhanced solubility - Enhanced stability	[89]
Preclinical	CUR	Liver fibrosis	- CCl_4_-induced liver fibrosis model in mice	- Curcumin loaded with chitosan-coated green nano silver particles	- Reduced liver enzyme levels - Prevented tissue destruction - Retained normal liver architecture	- Improved encapsulation - Increased permeability - Increased intrahepatic bioavailability - Enhanced aqueous solubility	[90]
Preclinical	CUR	Liver fibrosis	- CCl_4_ induced liver damage study in Wistar rats	- Curcumin loaded with polycaprolactone	- Reduced deposition of fatty acid - Reduced number of fibrotic cells - Reduced liver enzyme levels	- Improved the permeability - Enhanced cellular uptake	[91]
Preclinical	CUR	Fibrosis	- Thioacetemide-intoxicated rats	- Commercially available nanocurcumin (no further information is provided)	- Hepatoprotective and anti-inflammatory effect	- Less oxidative stress - Improved liver biochemistry (ALT, AST, ALP) - Improved lobular architecture and disappearance of inflammation and cholestasis	[92]

CUR, curcumin; HCC, hepatocellular carcinoma; PLGA, poly(lactic-co-glycolic acid); C210, (1E,6E)-4-(4-hydroxy-3-methoxybenzyl)-1,7-bis(3,4,5-trimethoxy phenyl)hepta-1,6-diene- 3,5-dione); CU-PTX-LNP, a lipid nano platform for coloading encapsulating curcumin and paclitaxel at ratios of 2:1–80:1 (*w*/*w*); Cur@β-CD-PEG-Chol, Cur-loaded nanomicelles developed by the conjugation of β-cyclodextrin (β-CD) and cholesterol onto both ends of the poly(ethylene glycol); IONs@Cur, curcumin capped iron oxide nanoparticles; Gal-Mor-LPs, galactose–morpholine-modified liposomes loaded with curcumin; CCl_4_, carbon tetrachloride; CeO_2_@SiO_2_, cerium oxide–silica nanoparticles; PAMAM-Gal, polyamidoamine dendrimers anchored to galactosamine; DEN, diethylnitrosamine; PAC, curcumin-loaded pullulan acetate nanoparticles; C-SLNs, curcumin-loaded solid lipid nanoparticles; NAFLD, nonalcoholic fatty liver disease.

## Abbreviations

CAP	Controlled attenuation parameter
ESD	End-stage liver disease
CLD	Chronic liver disease
HCC	Hepatocellular carcinoma
WHO	World Health Organization
JAK	Janus kinase
EMT	Epithelial mesenchymal transition
COX-2	Cyclooxygenase-2
PGE2	Prostaglandin E synthase 2
EGFR	Epidermal growth factor receptor
PI3K	Phosphatidylinositol 3-kinase
AKT	Protein kinase B
AMPK	AMP-activated protein kinase
LDL	Low-density lipoprotein
PPAR γ	Peroxisome proliferator-activated receptor gamma
ACAT	Acyl-CoA: cholesterol acyltransferase
DNL	De novo lipogenesis
NAFLD	Nonalcoholic fatty liver disease
ChREBP	Carbohydrate response element binding protein
SREBP1c	Sterol regulatory element binding protein
FFA	Free fatty acid
PLGA	Polylactic-co-glycolic acid
CAPs	Controlled attenuation parameters
SLD	Steatotic liver disease
ASGPR	Asialoglycoprotein receptor
MRT	Mean residence time
MASH	Metabolic dysfunction-associated steatohepatitis
NF-κB	Nuclear factor kappa B
PBMC	Peripheral blood mononuclear cell
PLGA	Polylactide co-glycolide
α-SMA	α-smooth muscle actin
STAT3	Signal transducer and activator of transcription-3
TME	Tumor microenvironment
VEGF	Vascular endothelial growth factor
CAF	Cancer-associated fibroblasts
TACE	Transarterial chemoembolization therapy
CurDD	Curcumin diethyl disuccinate
CXCR4	C-X-C chemokine receptor type 4
MACs	Monocarbonyl analogs of curcumin
PLGA	Polylactic-co-glycolic acid
Gal-Mor-LPs	Galactose–morpholine modified liposomes
IONs@Cur	Curcumin-capped iron oxide nanoparticles
CCl_4_	Carbon tetrachloride
CeO_2_@SiO_2_	Cerium oxide-silica nanoparticles
CSLNs	Curcumin-loaded solid lipid nanoparticles
PAMAM-Gal	Polyamidoamine dendrimers anchored to galactosamine
DEN	Diethylnitrosamine
PAC	Pullulan acetate nanoparticles
C-SLNs	Curcumin-loaded solid lipid nanoparticles
aHSC	Activated hepatic stellate cells
IHC	Immunohistochemistry
ASGPR	Asialoglycoprotein receptor
MASLD	Metabolic dysfunction-associated steatotic liver disease
MASH	Metabolic dysfunction-associated steatohepatitis
ALD	Alcoholic liver disease
